# Temporal Ordering of Dynamic Expression Data from Detailed Spatial Expression Maps

**DOI:** 10.3791/55127

**Published:** 2017-02-09

**Authors:** Charlotte S.L. Bailey, Robert A. Bone, Philip J. Murray, J. Kim Dale

**Affiliations:** ^1^The Danish Stem Cell Center (DanStem), University of Copenhagen; ^2^Division of Mathematics, University of Dundee; ^3^Division of Cell and Developmental Biology, College of Life Sciences, University of Dundee

**Keywords:** Developmental Biology, Issue 120, Notch, segmentation, Delta, chick, mouse, embryo, oscillations

## Abstract

During somitogenesis, pairs of epithelial somites form in a progressive manner, budding off from the anterior end of the pre-somitic mesoderm (PSM) with a strict species-specific periodicity. The periodicity of the process is regulated by a molecular oscillator, known as the "segmentation clock," acting in the PSM cells. This clock drives the oscillatory patterns of gene expression across the PSM in a posterior-anterior direction. These so-called clock genes are key components of three signaling pathways: Wnt, Notch, and fibroblast growth factor (FGF). In addition, Notch signaling is essential for synchronizing intracellular oscillations in neighboring cells. We recently gained insight into how this may be mechanistically regulated. Upon ligand activation, the Notch receptor is cleaved, releasing the intracellular domain (NICD), which moves to the nucleus and regulates gene expression. NICD is highly labile, and its phosphorylation-dependent turnover acts to restrict Notch signaling. The profile of NICD production (and degradation) in the PSM is known to be oscillatory and to resemble that of a clock gene. We recently reported that both the Notch receptor and the Delta ligand, which mediate intercellular coupling, themselves exhibit dynamic expression at both the mRNA and protein levels. In this article, we describe the sensitive detection methods and detailed image analysis tools that we used, in combination with the computational modeling that we designed, to extract and overlay expression data from distinct points in the expression cycle. This allowed us to construct a spatio-temporal picture of the dynamic expression profile for the receptor, the ligand, and the Notch target clock genes throughout an oscillation cycle. Here, we describe the protocols used to generate and culture the PSM explants, as well as the procedure to stain for the mRNA or protein. We also explain how the confocal images were subsequently analyzed and temporally ordered computationally to generate ordered sequences of clock expression snapshots, hereafter defined as "kymographs," for the visualization of the spatiotemporal expression of Delta-like1 (Dll1) and Notch1 throughout the PSM.

**Figure:**
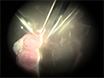


## Introduction

Somites are the first segments formed in the elongating body axis in developing vertebrate species and are the precursors of the spine, ribs, and dermis tissue, as well as of muscle and endothelial cells. During somitogenesis, epithelial somites form from the unsegmented presomitic mesoderm (PSM) (reviewed in Reference 1). This process is regulated by the "segmentation clock," which consists of a network of oscillatory genes and proteins, mostly belonging to the Notch signaling pathway. The segmentation clock consists of various negative feedback loops, which enable the pulsatile production of Notch activity within a single cell^2^ (reviewed in References 3 - 6). While the intracellular method of oscillation is well characterized, it is still largely unknown how these oscillations are coordinated across the PSM tissue. It has been recently shown, through both experimental and theoretical studies, that these oscillations are essential to the process of somitogenesis and that the Notch pathway plays a crucial role in the process of both segmentation and oscillatory gene expression^7^,^8^. However, it has been widely reported that Notch receptor 1 (Notch1) and Delta-like ligand (Dll)-1 have static gradients in the PSM^9^,^10^,^11^.

We hypothesized that Notch-dependent oscillations of the PSM segmentation clock depend upon the periodic activation of the main Notch pathway receptor and ligand, Notch1 and Dll1, respectively, across the mouse PSM. The conclusions of previous studies that reported a static rostral-caudal gradient of these proteins were due, we predict, to a lack of sensitivity in immunostaining techniques. They were therefore unable to detect low-level fluctuations of Dll1 and Notch1 in the caudal PSM.

We have devised a method to more closely examine these factors, combining experimental data with mathematical modeling to predict a mechanism by which the oscillations of the proteins of clock components are coordinated across the PSM^12^.

The overall goal of this method is to detect and quantify low-level, dynamic protein expression in the PSM and to map the expression profiles of proteins of interest according to the expression of the known clock gene, *Lunatic fringe *(*Lfng*). Since one cycle of the segmentation clock in the mouse embryo takes 2 h to complete, various samples are required to build a complete spatiotemporal profile of Dll1 and Notch1 protein expression during one *Lfng* oscillation in the PSM. We have thus developed this protocol to allow for the high-throughput detection of low-level protein expression in whole-mount, contralateral PSM explants. However, this technique can also be useful for studies that aim to characterize low-level protein dynamics within any embryonic tissue that can be split into contralateral halves.

## Protocol

All experiments were performed under project license number 6004219 in strict adherence to the Animals (Scientific Procedures) Act of 1986 and the UK Home Office Codes of Practice for the use of animals in scientific procedures.

### 1. PSM Explant Dissection

1. Obtain the tail tissue from embryos produced by the timed mating of wild-type (CD1) mice^13^. Briefly, at embryonic day (E) 10.5, euthanize the pregnant donor mouse in a carbon dioxide chamber. Harvest the uterine horn and place it into 1x sterile phosphate-buffered saline (PBS) solution in accordance with Home Office License procedures or equivalent local rules. Transfer the uterine horn to a tissue culture dish containing fresh, sterile PBS. Perform all subsequent dissection steps in this solution.
2. Under a stereomicroscope, cut the thick muscular membrane of the uterine horn using curved scissors and extract each embryo carefully using fine forceps. Take care to ensure that the tail tissue is not damaged in this process. Using curved scissors and fine forceps, dissect away the amniotic sac from each embryo, taking care not to damage the embryo.
3. Use either a surgical needle or curved scissors to harvest the tail tissue of each embryo by cutting the embryo posterior to the rear limb buds.
4. Balance the tail tissue ventral-side down using both forceps and a needle. Generate pairs of PSM explants from each embryonic tail by dissecting the tail tissue into two halves along the midline; perform a gentle rocking motion with a needle. Ensure that the neural tube, notochord, and PSM tissue are equally divided between the two explants.
5. Pipette each contralateral PSM explant onto the underside of a 35-mm plastic culture dish lid in a small volume of pre-warmed (37 °C) culture medium (DMEM-F12 + 0.1% L-glutamine substitute supplemented with 10% fetal calf serum, 10 nM human Bfgf, and 1% penicillin/streptomycin).
6. Place the dish on the top of the lid and quickly invert it so that the PSM tissue is suspended from the lid in a "hanging drop" of medium. Culture the PSM explants in a humidified chamber at 37 °C for 1 - 2 h.
7. Transfer pairs of PSM explants to the individual wells of a 24-well tissue culture plate. Incubate in 4% paraformaldehyde in PBS for 1 h at room temperature (RT) or 4 °C overnight (O/N). **CAUTION**: Paraformaldehyde is toxic, and appropriate safety measures must be taken when working with this solution. NOTE: Perform all subsequent washing and incubation steps in a 24-well tissue culture plate.
8. Wash the sample wells in PBS at RT on a rocking platform, using a fine plastic Pasteur pipette to exchange the PBS solution on the samples for fresh PBS 3 - 4 times. Process one PSM explant from each pair using immunohistochemistry (step 2) and the other using fluorescent *in situ* hybridization for a known clock gene (step 3).

### 2. Immunohistochemistry of PSM Explants

1. Wash one PSM explant from each embryonic pair generated in step 1 in 2% Triton X-100 in PBS for 1 h at RT on a rocking platform, and then rinse samples briefly in PBS. Replace the PBS on the samples with blocking solution (2% bovine serum albumin (BSA) and 10% normal goat serum (NGS) in PBS + 0.1% Tween-20) and incubate O/N at 4 °C on a rocking platform. NOTE: All subsequent washes and incubation steps in this section must be performed at RT on a rocking platform, unless otherwise stated. Wash solutions can be easily changed using a fine-tipped plastic or glass Pasteur pipette.
2. Dilute the desired primary antibody/antibodies in working buffer (0.1% BSA, 0.3% NGS, and 0.2% Triton X-100 in PBS). In this example, dilute the Dll1 and Notch1 antibodies 1:25 in working buffer. NOTE: Optimization will be required to determine the appropriate dilution factor required in this step if alternative antibodies are used.
3. Incubate explants in the antibody solution for 3 - 5 days at 4 °C on a rocking platform. Be sure to include some samples with working buffer containing no primary antibody to act as secondary antibody controls.
4. Recover the primary antibody solution in a 1.5-mL storage tube using a pipette and store it at 4 °C. NOTE: Recovered primary antibody can be used several times, depending on the antibody used.
5. Perform 2 washes of the samples for 5 - 10 min each in PBS, followed by 3 washes for 10 min each in 2% Triton X-100 in PBS at RT on a rocking platform.
6. Dilute fluorescently labeled secondary antibody/antibodies (epitope-matched to the primary antibody/antibodies used) in working buffer. Optionally, add 20 µg/mL Hoechst 33342 to this solution to counterstain the nuclei. NOTE: Optimization may be required to determine the appropriate dilution factor required in this step. In this example, a dilution factor of 1:400 was typically used.
7. Centrifuge the secondary antibody solution for 10 min at 16 x g to prevent the formation of antibody aggregates. Add 250 - 500 µL of the secondary antibody solution to each sample well, taking care not to use the last few microliters of the solution, which may contain antibody aggregates.
8. Cover the sample plate with tin foil to minimize light exposure and incubate the samples in the secondary antibody solution for 3 - 5 days at 4 °C in the dark.
9. Prior to sample mounting, wash the samples twice for 10 min each in 0.1% Tween-20 in PBS (PBST) and once for 5 min in PBS at RT on a rocking platform (see step 4).

### 3. Fluorescent *In Situ* Hybridization (FISH) of PSM Explants

1. If stored in an alternative vessel, transfer the remaining contralateral PSM explants to the individual wells of a 24-well tissue culture plate.
2. Wash the samples for 10 min in 50% ethanol in PBST, and then perform 2 washes for 10 min each in 100% ethanol on a rocking platform at RT to dehydrate the tissue. NOTE: All subsequent washes and incubation steps in this section must be performed at RT on a rocking platform, unless otherwise stated.
3. Rehydrate the tissue by washing for 10 min in 50% ethanol in PBST, followed by washing twice for 5 min each in PBST. NOTE: Steps 3.2 and 3.3 are necessary fixation steps required for this protocol and cannot be omitted.
4. Incubate the samples with 10 µg/mL proteinase K in 0.1% Tween-20 in PBS (PBST) for 5 min without agitation. Quickly remove the proteinase K and rinse the samples briefly with PBST before post-fixing the tissue for 30 min in 4% formaldehyde + 0.1% glutaraldehyde in PBST. **CAUTION:** Both formaldehyde and glutaraldehyde are toxic, and appropriate safety measures must be taken when working with these solutions. NOTE: The following washing and incubation steps involving 50% and 100% hybridization mixes (steps 3.6 - 3.9) should be performed without agitation.
5. After washing the samples twice for 10 min each in PBST, wash the samples once in 50% hybridization mix (suitable for intronic probes: 50% formamide, 5x saline-sodium citrate (SSC), 5 mM EDTA, 50 µg/mL tRNA, 0.2% Tween-20, 0.1% SDS, and 100 µg/mL heparin) in PBST prepared at RT. Incubate the samples in this solution for 10 min at 65 °C without agitation.
6. Rinse the samples twice with pre-warmed (65 °C) hybridization mix before incubating the samples in hybridization mix for ≥ 2 h (up to 48 h) at 65 °C (longer incubation times improve the resulting signal-to-noise contrast). Remove the hybridization mix from the previous step, and replace it with 0.25 - 0.5 mL of pre-warmed (65 °C) hybridization mix containing a digoxigenin (DIG)-labeled anti-sense RNA probe against a known segmentation clock component. NOTE: For example, an intronic Lunatic fringe (*Lfng(i)*) probe was used at a concentration of 20 µL/mL to detect nascent *Lfng* mRNA. The dilution used in this step is probe-dependent and will require optimization.
7. Seal the plate using sticky tape to prevent evaporation and incubate the samples in the probe solution for two nights at 65 °C.
8. Using a fine-tipped plastic Pasteur pipette, recover the probe for reuse and store it at 20 °C. Rinse the samples twice with pre-warmed (65 °C) post-hybridization mix (50% formamide, 0.2% Tween-20, and 1x SSC) before washing the samples two more times for 20 min each at 65 °C in pre-warmed post-hybridization mix.
9. Wash the samples for 15 min at 65 °C in pre-warmed 50% hybridization mix in 0.1% Tween-20 in Tris-buffered saline solution (TBST). Rinse the samples twice with TBST before washing for 30 min at RT in TBST on a rocking platform.
10. Pre-incubate the explants in blocking solution (TBST + 2% blocking buffer reagent (BBR) + 20% heat-treated goat serum) for a minimum of 2 h. Replace this solution with fresh blocking solution containing a 1:200 dilution of horseradish peroxidase (HRP)-conjugated anti-digoxigenin antibody. Incubate the samples O/N at 4 °C.
11. After the antibody incubation, rinse the samples 3 times with TBST at RT and transfer them to individual wells of a new 24-well tissue culture plate. Wash the explants with TBST 3 times for 1 h each.
12. At this point, transfer the samples into 0.5-mL storage tubes or the individual wells of a 48-well tissue culture plate to reduce the required volume of the Tyramide signal amplification (TSA) detection reagents in the following steps.
13. Incubate samples in TSA amplification buffer (see the reagents list) at RT for 1 min without agitation using as small a volume as possible, ensuring that the samples are fully immersed in the solution.
14. Add TSA reagent (see the reagents list) to the sample amplification buffer at a dilution of 1:50. Quickly mix the solution until the TSA reagent is evenly distributed, cover the plate or tubes in tin foil, and incubate the samples for 60 - 90 min in the dark.
15. Remove the TSA amplification solution and wash the samples in TBST 3 times for 5 min each. Transfer the explants back to a 24-well tissue culture plate to increase the wash volume and incubate the samples in 1% hydrogen peroxide in TBST for 1 h. Wash the samples with TBST 3 times for 5 min each, and then twice for 5 min each with PBST prior to sample mounting (see step 4).

### 4. Sample Preparation for Imaging

1. Prepare one charged adhesion glass slide for each explant pair by adding 0.12-mm thick imaging spacers, which prevent the samples from being crushed by the addition of a coverslip. Remove the adhesive liner from one surface of a spacer and place it adhesive-side down onto a glass slide, pressing firmly to seal the spacer to the slide. NOTE: For the remaining steps, endeavor to keep the samples in low light or in darkness to avoid photobleaching. Pipette explant pairs onto a prepared slide using a glass Pasteur pipette within the center of the spacer, ensuring that the dissected side of the explant faces the slide. Arrange contralateral pairs of explants side by side.
2. Remove as much liquid as possible from the slide using a glass Pasteur pipette and wick off any residual moisture surrounding the samples using a piece of folded low-lint tissue paper.
3. Allow the samples to adhere to the slide for 45 - 60 s, until the tissue begins to appear sticky and translucent. During this time, remove the remaining adhesive liner from the spacer using forceps. Do not allow the samples to dry out.
4. Add a large drop of dual-function mountant and clearing solution (0.5% p-phenylenediamine and 20 mM Tris, pH 8.8, in 90% glycerol) to the samples within the center of the spacer. NOTE: This solution turns brown/black when permitted to oxidize.
5. Carefully place a circular coverslip (no. 1.5) across the samples, ensuring that the mountant is evenly distributed and that all edges of the coverslip make contact with the spacer. Place the cover-slipped slide upside down onto some low-lint tissue paper.
6. Press down firmly to ensure that the coverslip fully adheres to the spacer and that any excess mountant is removed. Repeat until no more mountant blots the paper.
7. Clean and label the slide(s) appropriately, and store them in the dark until imaging, short-term at -20 °C or long-term at -80 °C. After removing the slides from storage, allow them to fully thaw before imaging.
8. Image the mounted samples using a confocal microscope with tiled acquisition and a high magnification objective. Image the explant pairs using a 40X oil immersion objective at 4-µm z-intervals using 488-nm, 568-nm and 647-nm laser lines to excite the green, red, and far-red fluorophores, respectively, employed for protein and mRNA detection in this study^12^. NOTE: Tiled images were stitched post-acquisition to form a single image for analysis.

### 5. Post-acquisition Image Analysis

1. Use image analysis software to define a region of interest within the PSM of each experimental sample.
  1. To quantify expression levels, subtract background and threshold images to the level of a no-primary control sample prior to subsequent quantification. Define an origin, an axis, and a unit length for each sample.
2. Calculate the fluorescence intensity as a function of position along the normalized rostro-caudal axis for each of the M samples ^12^. After normalizing the intensity plots, place the intensity profiles side by side and obtain an intensity matrix *f(i,j)* that describes the intensity at the *i*^th^ spatial position in the *j*^th^ sample.

### 6. Temporal Ordering of Samples

1. To infer temporal ordering of a known clock component, define its intensity matrix. Then, rearrange the columns of the intensity matrix so as to obtain a temporally periodic pattern. To do this, define the function 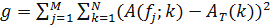 where *A(f_j_;k)* represents the autocorrelation function of the *j*^th^ column of *f* and *A_T_* is a target autocorrelation function, chosen to enforce the temporal periodicity of the pattern, given by 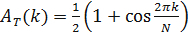
2. Use the Metropolis-Hastings (or another minimization algorithm)^12^ to identify the order of the samples that minimize the function *g*. Thus, determine the order of the M samples that maximizes the temporal periodicity of a known clock component.
3. Using the inferred temporal ordering of the M samples, construct an ordered kymograph for the expression pattern in the partnered channel^12^.

## Representative Results

This protocol permits the visualization of the spatiotemporal profile of a protein of interest alongside clock gene transcription in the mouse PSM^12^. For example, Dll1 (**Figure 1A-C**) and Notch1 (**Figure 1D-F**) protein expression are shown to oscillate out of synchrony with the nascent transcription of the Notch-regulated segmentation clock gene *Lfng*. Quantification of Dll1, Notch1, and *Lfng(i)* signal intensity in relation to the antero-posterior (AP) axis of the PSM (**Figure 1G**) reveals clear oscillatory expression dynamics for these targets (**Figure 1H-J**). The spatiotemporal profile of Dll1 and Notch1 protein expression throughout the clock cycle are clearly visualized and quantified using this protocol through the post-acquisition image analysis of high-resolution fixed tissue image data.


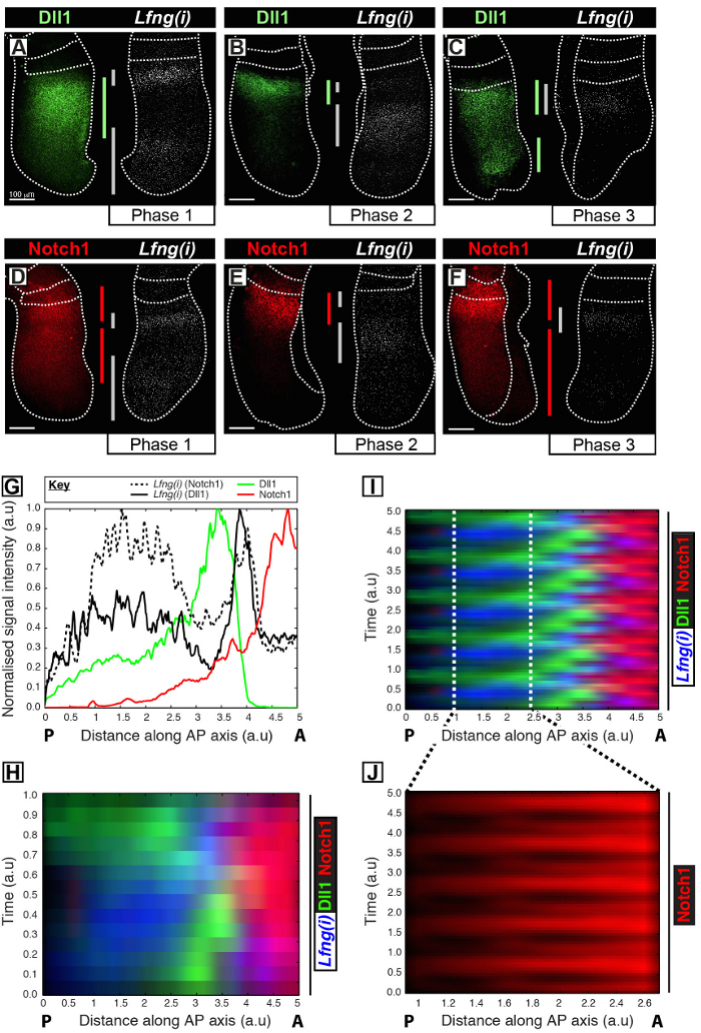
**Figure 1: Spatio-temporal Visualization and Quantification of Dll1 and Notch1 Protein Expression Dynamics.** (**A-F**) Pairs of explants from six E10.5 embryos (**A-F**) showing the spatial distribution of Dll1 protein (**A-C**) or Notch1 protein (**D-F**) in one half alongside the detection of *Lfng* pre-mRNA (*Lfng(i)*) in the corresponding contralateral half of each pair. Panels are arranged according to Phase 1 (**A **and** D**), Phase 2 (**B **and** E**), and Phase 3 (**C** and** F**) of the segmentation clock cycle, as determined by the spatial profile of *Lfng(i)* expression. The extent of the expression domains for Dll1 (green), Notch1 (red), and *Lfng(i)* (gray) along the antero-posterior axis of the PSM have been demarcated by color-coded bars. The dotted lines demarcate the positions of the most recently formed somite(s), the outer edges of the PSM, and the adjacent neural tissue (**C** and **E**). Scale bars (bottom left of each panel, **A-F**) represent 100 µm. (**G**) An example intensity plot depicting the axial variation in signal intensity across the PSM. The data is plotted from two explant pairs showing *Lfng* pre-mRNA (black hashed line) in one explant compared to Notch1 protein (red) in the contralateral explant (Embryo 1), as well as *Lfng* pre-mRNA (black solid line) in another explant compared to Dll1 protein (green) in the contralateral explant (Embryo 2). Measured signal intensity (y-axis) is plotted against axial position (x-axis; anterior PSM [A] to the right and posterior PSM [P] to the left). (**H**) A kymograph showing the spatial distribution of Dll1, Notch1, and *Lfng(i)* across numerous PSMs. Each row of the kymograph represents the signal intensity of an individual PSM explant. Rows are arranged in temporal sequence according to the spatiotemporal distribution of *Lfng* pre-mRNA (**I**) The spatiotemporal distribution of Dll1, Notch1, and *Lfng(i)* through multiple clock oscillations is simulated by the periodic extension of the data shown in (**H**), highlighting the oscillatory nature of Dll1 and Notch1 expression dynamics. (**J**) Pulsatile Notch1 protein expression in the caudal PSM is highlighted by magnification of the region demarcated in the virtual kymograph shown in (**I**). Modified from Reference 12. Please click here to view a larger version of this figure.


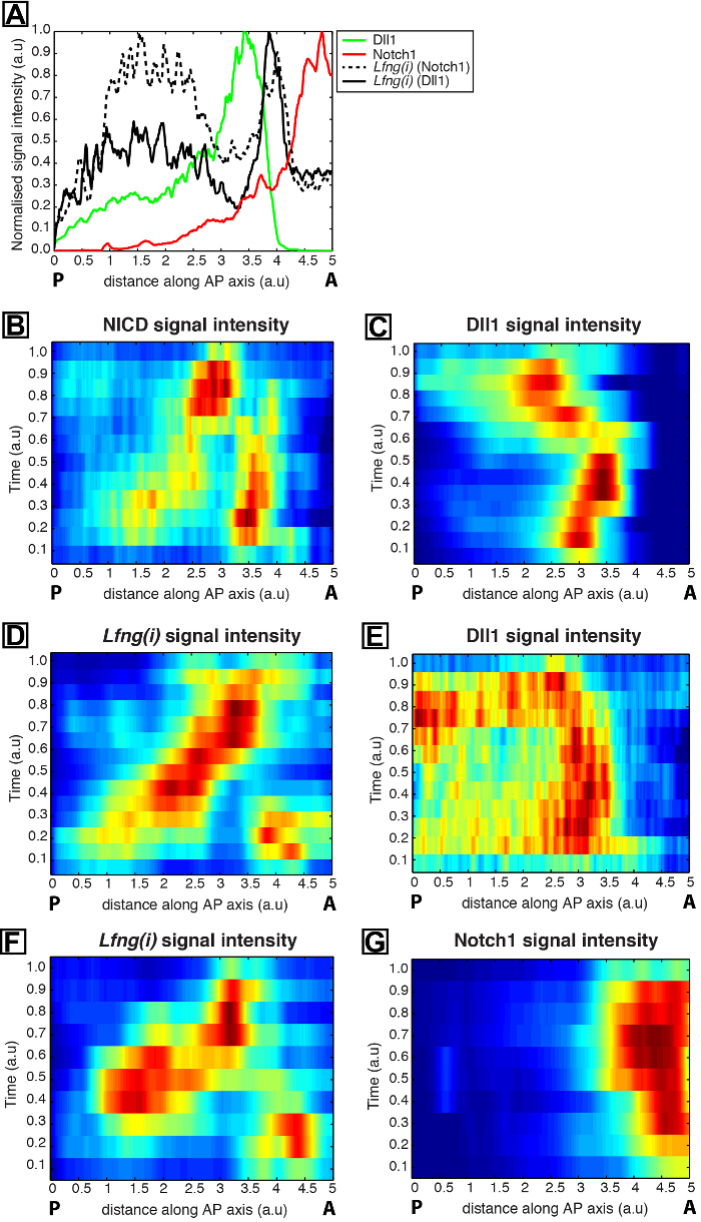
**Figure 2: Quantification of the Spatio-temporal Dynamics of Dll1 and Notch1 Protein Expression. **(**A**) An example intensity plot depicts axial variation in signal intensity across the PSM. Data plotted from two explant pairs showing *Lfng* pre-mRNA (black hashed line) in one explant compared to Notch1 protein (red) in the contralateral explant half, as well as *Lfng* pre-mRNA (black solid line) in a half explant from a second tail compared to Dll1 protein (green) in the contralateral explant half of the second tail. Measured intensities (y-axis) are plotted against axial position (x-axis; rostral [A] to the right and caudal [P] to the left). (**B-H**) Kymographs show the spatial distribution of Notch1, Dll1, NICD, and *Lfng(i)* across numerous PSMs. (B and C) NICD (B) and Dll1 (**C**) expression in PSM sections; (**D** and **E**) *Lfng(i)* (D) and Dll1 (**E**) in contralateral explant halves; (**F** and **G**) *Lfng(i)* (**F**) and Notch1 (**G**) in contralateral explant halves. From Reference 12. Please click here to view a larger version of this figure.

## Discussion

### Critical Steps within the Protocol

The present protocol describes a sensitive method to perform the quantitative analysis of low-level protein expression and oscillatory dynamics in E10.5 mouse PSM explants. A robust protocol for both immunohistochemistry and fluorescent *in situ* hybridization (FISH) is followed by high-resolution whole-mount confocal imaging, and then by image analysis and temporal segmentation of kymographs to generate a spatiotemporal map of protein expression across the PSM. A high signal-to-noise ratio in protein and mRNA detection is essential to ensure the success of this technique. Care must be taken to thoroughly exchange all solutions effectively during the wash steps and to maintain the temperature of the 65 °C washes in the relevant stages of step 3. It is most advantageous to take the time to source efficacious antibodies and RNA probes against the targets of interest and to test these reagents thoroughly on whole-mount samples prior to starting this protocol.

### Modifications and Troubleshooting

The main issues that may be encountered when performing this protocol arise from poor signal detection strength and quality. This is largely dependent upon the efficacy of the antibodies or RNA probes used for the immunohistochemistry or FISH steps in the protocol, respectively. A number of different steps may require optimization before adequate signal detection is achieved. One common cause for poor signal detection is improper fixation; it is imperative that either fresh PFA or PFA stored at 4 °C for no longer than one week is used to fix the samples. The length of fixation may also require optimization, depending on the antibody or RNA probe used. For antibodies, it is advised to follow the manufacturer's instructions where possible, while for RNA probes, we advise the consultation of the published literature.

In this study, we used an RNA probe that specifically detects the pre-mRNA of the clock gene *Lfng*. Due to its relative lack of abundance, detection of *Lfng* pre-mRNA requires a long period of incubation with the probe in hybridization mix containing 5x saline-sodium citrate (SSC) for good signal detection. The same conditions may apply to other probes that detect weakly expressed mRNAs, but in our experience, the detection of more stable mRNA targets may require a shorter probe hybridization step and lower SSC concentrations in the hybridization mix (*e.g.,* 1.3x SSC). For both immunohistochemistry and FISH, the protocol must first be optimized on whole embryos, and the optimal concentration of antibody or probe must be determined empirically.

### Limitations of the Technique

As mentioned above, the success of this technique is highly dependent upon the quality of the protein and mRNA detection. We have outlined several suggestions as to how protein and mRNA detection can be improved, but in the absence of high-quality fluorescent signal detection, there is no way the experiment can proceed. The number of protein targets that can be analyzed in each tissue sample is limited by the spectral resolution of the confocal microscope and by the epitopes of the antibodies used. In this study, we were able to use up to three epitopes for protein detection alongside a DNA stain on each sample^12^. This protocol only permits the detection of one mRNA target, although current alternative methods could be employed to increase this to up to three targets^14^.

### Significance of the Technique with Respect to Existing/Alternative Methods

The method described here provides a sensitive technique to detect low-level protein fluctuations in whole-mount PSM explants. The quantification of these dynamics is possible by performing FISH for a known clock gene in corresponding contralateral explants. A library of kymographs is generated that can be organized over one segmentation clock cycle, highlighting the spatiotemporal expression dynamics of a target of interest within this time frame. A key difference in this technique over others is the use of computational automation to temporally order large data sets, which permits the spatiotemporal expression dynamics of novel clock components to be analyzed in an unbiased manner. For example, this technique provided insight into how Dll1 and Notch1 proteins and their oscillations are co-regulated across the entire PSM. Alternative methods in this context have also relied upon immunostaining, but they did not detect the small fluctuations in Dll1 and Notch1 protein levels in the caudal PSM that were evident using this method. Instead, they reported a steady gradient of expression that is strongest in the rostral region^9^,^10^,^11^. This could be due to the fact that this protocol has a lengthier primary antibody incubation period (3 - 5 days, as opposed to overnight), which may be required to detect lower levels of protein. As the levels of Dll1 and Notch1 expression are relatively high in the rostral PSM, this may have influenced the authors to image the samples at a lower exposure setting than would be necessary to detect the caudal protein expression. One further potential discrepancy arises from the use of unfixed tissue in the study by Chapman *et al.*, in which the transitory expression of Dll1 and Notch1 in the caudal PSM may have been less well-preserved^9^.

### Future Applications or Directions after Mastering the Technique

Once this protocol has been mastered, high-throughput expression analysis can be performed for any protein of interest in the PSM. PSM explants generated from several mouse litters can be processed at once to generate the high sample number necessary for analysis. Although we have only used wild-type embryos in these studies, it is possible to perform this analysis using genetically modified embryos in order to assess the importance of one or more factors on protein expression dynamics. Beyond the PSM, this protocol can be adapted to other systems that are composed of two contralateral halves and can be used to sensitively detect low-level protein expression and oscillatory dynamics. One example for which this protocol could be adapted is the study of dynamic protein expression in the mouse neural tube, since contralateral halves could be generated and cultured, and Notch activity has been shown to be both present and important for patterning^15^. We encourage other groups to adapt this protocol to other systems and to provide feedback for future improvement.

## Disclosures

The authors have nothing to disclose.
